# Shape configuration of mental targets representation as a holistic measure in a 3D real world pointing test for spatial orientation

**DOI:** 10.1038/s41598-023-47821-2

**Published:** 2023-11-22

**Authors:** J. Gerb, T. Brandt, M. Dieterich

**Affiliations:** 1 German Center for Vertigo and Balance Disorders, Ludwig-Maximilians University, Munich, Germany; 2 Graduate School of Systemic Neuroscience, Ludwig-Maximilians University, Munich, Germany; 3 Department of Neurology, Ludwig-Maximilians University, Munich, Germany; 4 Hertie Senior Professor for Clinical Neuroscience, Ludwig-Maximilians University, Munich, Germany; 5 Munich Cluster for Systems Neurology (SyNergy), Munich, Germany

**Keywords:** Navigation, Perception, Spatial memory, Dementia, Neurodegeneration, Neurophysiology

## Abstract

Deficits in spatial memory are often early signs of neurological disorders. Here, we analyzed the geometrical shape configuration of 2D-projections of pointing performances to a memorized array of spatially distributed targets in order to assess the feasibility of this new holistic analysis method. The influence of gender differences and cognitive impairment was taken into account in this methodological study. 56 right-handed healthy participants (28 female, mean age 48.89 ± 19.35 years) and 22 right-handed patients with heterogeneous cognitive impairment (12 female, mean age 71.73 ± 7.41 years) underwent a previously validated 3D-real-world pointing test (3D-RWPT). Participants were shown a 9-dot target matrix and afterwards asked to point towards each target in randomized order with closed eyes in different body positions relative to the matrix. Two-dimensional projections of these pointing vectors (i.e., the shapes resulting from the individual dots) were then quantified using morphological analyses. Shape configurations in healthy volunteers largely reflected the real-world target pattern with gender-dependent differences (ANCOVA area males vs. females F(1,73) = 9.00, p 3.69 × 10^−3^, partial η^2^ = 0.10, post-hoc difference = 38,350.43, **p**_**bonf=**_3.69 × 10^−3^**, Cohen’s d 0.76, t 3.00). Patients with cognitive impairment showed distorted rectangularity with more large-scale errors, resulting in decreased overall average diameters and solidity (ANCOVA diameter normal cognition/cognitive impairment F(1,71) = 9.30, p 3.22 × 10^−3^, partial η^2^ = 0.09, post-hoc difference = 31.22, **p**_**bonf=**_3.19 × 10^−3^**, Cohen’s d 0.92, t 3.05; solidity normal cognition/cognitive impairment F(1,71) = 7.79, p 6.75 × 10^−3^, partial η^2^ = 0.08, post-hoc difference = 0.07, **p**_**bonf=**_6.76 × 10^−3^** Cohen’s d 0.84, t 2.79). Shape configuration analysis of the 3D-RWPT target array appears to be a suitable holistic measure of spatial performance in a pointing task. The results of this methodological investigation support further testing in a clinical study for differential diagnosis of disorders with spatial memory deficits.

## Introduction

Acquired deficits in spatial memory and orientation often indicate inceptive mild cognitive impairment or sensory deficits^1,2^. Numerous clinical and experimental tests of measuring spatial orientation have been proposed with various methodological advantages. Individual orientation and spatial skill levels scatter widely; they are dependent on a multitude of factors such as psychosocial elements, childhood hobbies, sex, age or cultural distinctions^3–5^. One common well-established every-day activity involving spatial orientation is pointing towards a target, e.g., for raising attention or awareness of its localization in a 3D-environment. In order to measure spatial performance in a clinical setting using a fast and simple test, we introduced a 3D-pointing paradigm (3D-real world pointing task, 3D-RWPT, Ref.^6^) using a previously optimized smartphone-based device^7^. The 3D-RWPT requires participants to point towards a memorized array of visual targets with the eyes closed and to update their internal map of the set of targets relative to changes in spatial body orientation after rotations in the yaw plane. These whole-body rotations were included in order to provide a labyrinthine stimulus, since vestibular disorders such as bilateral vestibular loss can impair spatial abilities^8,9^. Instead of simply calculating the differential angles between the real directions of the targets relative to the subject and the pointing directions^6,10^, in the current study we chose another approach for evaluation of the pointing performance using a *figure frame* (shape configuration of the target matrix) analysis. We introduced this metric in order to detect systematic changes of spatial performances that may go undetected in, e.g., single-point-deviation analyses. This kind of anlysis is based on classic cognitive psychology models of sensory processing architecture. The latter can be separated into three subsystems mediating peripheral, central, and sensorimotor processing programs: sensory input (i.e., the primary sensory organ information), central processing (i.e., the integration performed using various neuroanatomical pathways and cortical regions), and (motor) output systems^11^. In line with this terminology, we will use *primary figure* for the real-world geometrical configuration of the targets (in our case, the regular 9-dot matrix) perceived by the participant and *tertiary figure* for the overall test result, e.g., accuracy and angular deviation to each calibration^6^. By using a well-established intuitive motor output like finger pointing and detaching the pointing vectors from the real-world targets, in this study we assess the possibilities of *secondary figure* analysis, i.e., a semi-direct measurement of the underlying central shape configuration. Where classic spatial research focuses on e.g., accuracy in real-world interactions, and might compare participant navigation patterns in a ground-truth environment, the analysis of the secondary *figure frames* in theory allows for the investigation of participant-specific mental misrepresentations, systematic mislocations or spatial misconceptualisations. Importantly, the individual targets are reproduced by the participants in a randomized order; the underlying real-world shape is therefore initially disintegrated for the actual test and only “reassembled” through the figure frame analysis. In combination with, e.g., accuracy analysis or other measurements of the output system, this approach can potentially provide insight into the nature of spatial disturbances. Patients with visuospatial deficits due to parietotemporal degeneration might encode their spatial environment in an erroneous secondary *figure frame*, causing errors in real-world-interaction due to this skewed internal model. Importantly, the (e.g., motor) output aspects of the interactions might be performed accurately. Other diseases might primarily affect the (motor) implementation aspect of real-world interaction or, in case of sensory deficits, cause impairments of the primary *figure frame* (e.g., decreased visual acuity in macular degeneration^12^).

In this pilot study we compared the thus analyzed *figure frames* in healthy participants with special focus on female/male gender specific differences and the performance alterations observed in patients with unclassified cognitive impairment recruited in a dementia screening test. Our aim was not to test *figure frames’* differentiating power between different forms of dementia. Instead, we wanted to investigate the general feasibility of this additional shape configuration analysis. Thus far, morphological analysis of real-world targeted pointing performances has not been demonstrated.

## Methods

### Subjects

Two groups of participants were enrolled. The first group consisted of 56 healthy participants (28 female, mean age 48.89 ± 19.35 years) with scores higher than 26 points in the screening by the Montreal Cognitive Assessment (MoCA)-test^13^. The second group consisted of 22 patients (12 female, mean age 71.73 ± 7.41 years) with preferably mild cognitive impairment assessed by MoCA-testing testing (mean MOCA-score 22.92 ± 2.78; 24-25pts: n = 8, < 24pts: n = 14) or comparable neuropsychological test batteries as part of subsequent in-house diagnostics. All participants were recruited from the tertiary German Center for Vertigo and Balance Disorders where the MoCA was part of the routine investigation. Only those patients participated who had normal results in neurootological and neuroorthoptic examinations. While the MoCA test can only act as a screening tool and does not allow any inferences to the underlying disorders, the particular diagnoses were not a determinant of the current study. For this methodological investigation, we did not adapt MoCA-scores for age and sex of the participants^14^, but corrected for education level. Further diagnostics were recommended for patients with suspected cognitive impairment. Final diagnoses according to the respective diagnostic criteria were vascular encephalopathy (n = 8), frontotemporal dementia (n = 2), idiopathic Parkinson syndrome (n = 2), Alzheimer’s dementia (n = 1), normal pressure hydrocephalus (n = 1) and mixed dementia with epilepsy (n = 1). Three patients with only mild cognitive impairment had an as yet not further classified neurodegenerative disorder (mean MoCA 24.44 ± 0.94) while four patients (mean MoCA 23.75 ± 1.64) were lost to follow-up.

The Patient Health Questionnaire subsection 9 (PHQ-9, Ref.^15^) was used to rule out depression as a possible confounding factor. Handedness (for the pointing paradigm) was assessed using the Edinburgh handedness inventory^16^.

The data protection clearance and Institutional Review Board of the Ludwig-Maximilians-Universität München, Germany, approved the study (no. 094-10). The study was performed in accordance with the ethical standards laid down in the 1964 Declaration of Helsinki and its later amendments. All patients gave informed consent.

### 3D real world pointing test

**Figure 1:**
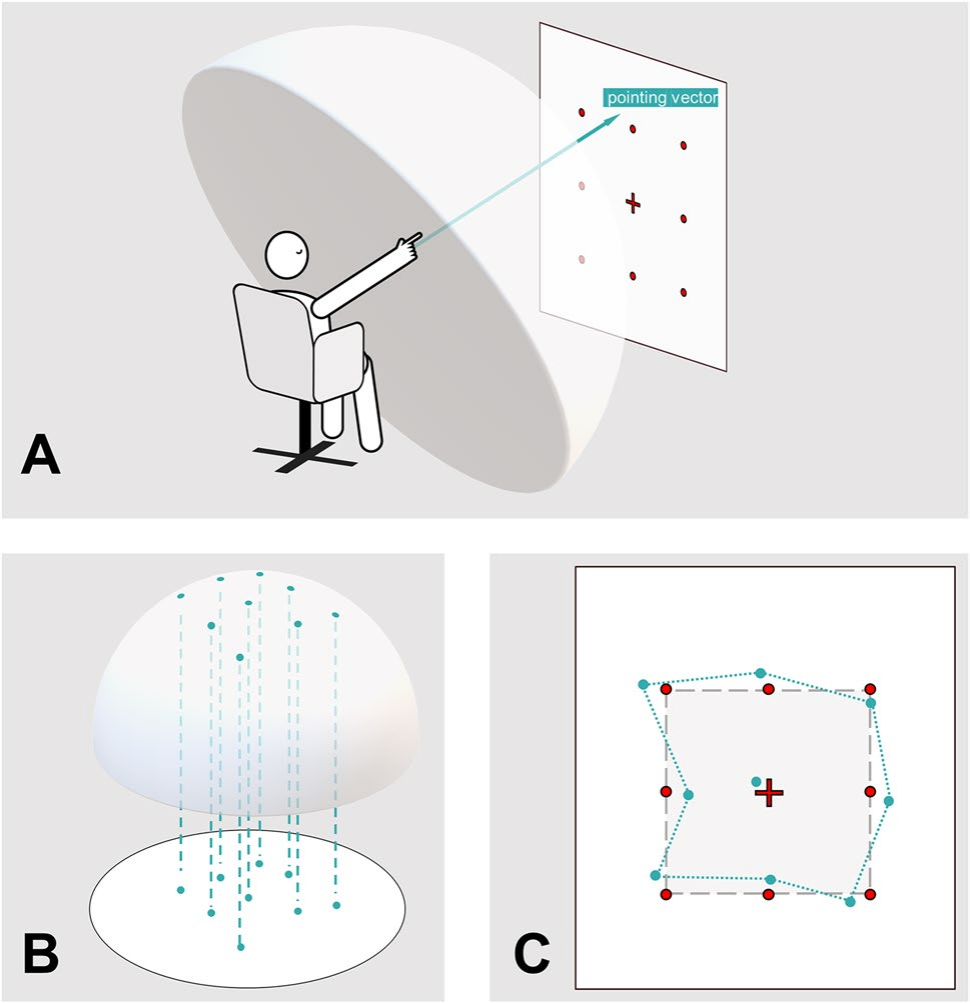
Depiction of the pointing task and *figure frame* creation steps. (**A**) After two sets of calibrations, the participant is given instructions to point towards different points of a 3 × 3 matrix with their eyes closed (petrol arrow) in randomized order, i.e., as singular dots rather than as a consecutive nine-dot-shape. The pointing vectors are recorded by the device (as described in Ref.^10^) as spherical coordinates (visualized here by a hypothetical half dome centered on the participants shoulder). This test was repeated five times: (1) in the initial position, (2) following a 90° rotation to the non-hand-dominant side performed with open eyes, (3) following a 90° rotation back to the starting position performed with closed eyes, (4) following a 90° rotation to the hand-dominant side performed with open eyes, and (5) following a 90° rotation back to the starting position performed with closed eyes. (**B**) The pointing vector’s intersections with the half dome can then be projected onto a 2D plane, creating the individual *figure frame* for each paradigm. (**C**) This unitless visual representation of the 3D pointing performance (petrol dotted line) can then be compared to the original target structure (3 × 3 matrix, grey rectangle and dark grey dashed line).

The clinical pointing task was performed using a pointing device attached at the participants forearm and testing setup from previous work and consisted of two calibration and five testing paradigms^5^. Calibration was performed with the eyes open and visual feedback available. Targets were marked with red 20 mm points on a white wall in a 3 × 3 matrix with a 100 cm distance between points. Participants were seated on a revolving chair with their eye level adjusted to match the center row of dots (Fig. 1). An eye-to-wall distance of 192 cm was chosen to ensure all targets were located in the near-peripheral field of vision (up to 30° radially outwards, Ref.^17^) when participants’ gaze was straight ahead on the central target; in the target matrix, neighboring points were separated by 27.5°, totaling to a target angular scope of 55° × 55° in azimuth and polar direction, respectively. For each task, a computerized voice from the device gave a randomized command, e.g., “top left” in either German or English. The subjects pointed towards the target with an extended arm and confirmed the measurement with a wireless Bluetooth dongle with the other hand; alternatively, the examiner could confirm each measurement. This calibration was repeated twice: once with a laser pointer attached to the device indicating the real-world trajectory of the participants arm (world-based calibration) and once without real-world visualization of the pointing vector; here, participants were instructed to visually align their index finger with the given target, i.e., align the retinotopic representation of target and finger ("*Peilen",* retinotopic calibration). If participants were unable to perform two calibration sets, the 3D-RWPT was aborted at this point. After calibration, the subjects were asked to point to the targets in randomized order without visual feedback (eyes closed) while facing straight ahead (1), after being passively 90° rotated to their “non-hand-dominant” side (i.e., towards the left side in our right-handed collective) with visual feedback available during rotation and pointing thereafter with their eyes closed (2), back in the initial position without visual feedback during rotation (3), after being passively rotated 90° to their “hand-dominant” side (here, towards the right side) with visual feedback available during rotation but eyes closed when pointing (4) and back to the initial target-facing position without visual feedback during rotation (5). The test instructions were to point to the individual dots; no instructions to remember or reproduce the overall shape or comparable cues were given. Each test was separated by a standardized pause of 30 s signaled in five second intervals using a notification sound. The rotations were performed by the examiner and took 1–2 s each, regardless of the participant’s cognitive status. For the tasks themselves, no time constrictions were given. On average, the whole test (including standardized breaks) typically took 7–10 min.

### Single point data analysis and figure frame creation

The pointing vectors from the 3D-RWPT can be used to calculate mean angular deviations in azimuth (≘horizontal) and polar (≘ vertical) plane between the two sets of calibrations and the five tasks, respectively as described in previous studies^6,10^. In this study, we further defined a 2D-projection of the raw pointing vectors as a depiction of the spatial pointing performance (without dimensional units) in order to assess the underlying mental representation of the target shape. Given the task’s angular scope of 55° between left and right or up and down, respectively, we chose to simply use a planar intersection of the unit sphere from which the azimuth and polar coordinates were derived since edge regions (which would undergo relevant compression effects) were not part of the 3D-RWPT. Pointing vectors closer to the rim of a hypothetical participant-centered half-dome would e.g., require stereographic projection. We will use the term *figure frame* for this representation. In our task with a rectangular equidistant dot matrix as a primary figure, the secondary figure resembles that rectangular shape. The center target was excluded to simplify geometrical shape analysis (Fig. 1).

#### *Figure frame* area calculation and shape analysis

In further shape analysis, for every participant seven paradigm-wise *figure frames* were plotted using ImageJ/FIJI^18^ on a 2000 pixel × 2000 pixel canvas: (1) world-based calibration, (2) retinotopic calibration, (3) initial reproduction, (4, 6) rotations towards non-hand-dominant and hand-dominant sides, (5, 7) post-rotation reproductions following rotation towards non-hand-dominant and hand-dominant sides. This step therefore creates seven irregular polygons per participant with distinct geometrical properties. We then used the MorphoLibJ-plugin^19^ for further morphological analysis, i.e., area (number of pixels), perimeter (Crofton method), circularity (ratio of area/squared perimeter), largest Feret diameter (maximum distance between all pairs of points belonging to the region) and Feret angle, and location of fitted boxes or ellipses, convexity (ratio of polygon area/convex hull area), average diameter, as well as radius and position of an inscribed disc for each single *figure frame* in all single participants. Other parameters were calculated, but eventually discarded since they resulted in inaccurate measurements in cases of large-scale errors (e.g., Euler number for connected components being artificially high in case of long and thin outliers; tortuosity, geodesic elongation or diameter using Chamfer distances propagation being infinite due to their MorphoLibJ-implementation).

### Statistical analyses

After data collection, all data was irreversibly anonymized for data analyses and processed using Microsoft^®^ Excel (Version 2022) and JASP (Version 0.16.4^20^). Mean values and standard deviation were taken for continuous variables and absolute and relative frequencies for categorical variables. Statistical inference using Pearson’s r and Spearman’s rho was tested and one-way analysis of variance (ANCOVA) performed testing for each pointing paradigm when comparing the subgroups (post-hoc-testing using 1000 bootstraps, Bonferroni-correction and effect size estimation with partial η^2^, all analyses performed in JASP).

## Results

**Figure 2:**
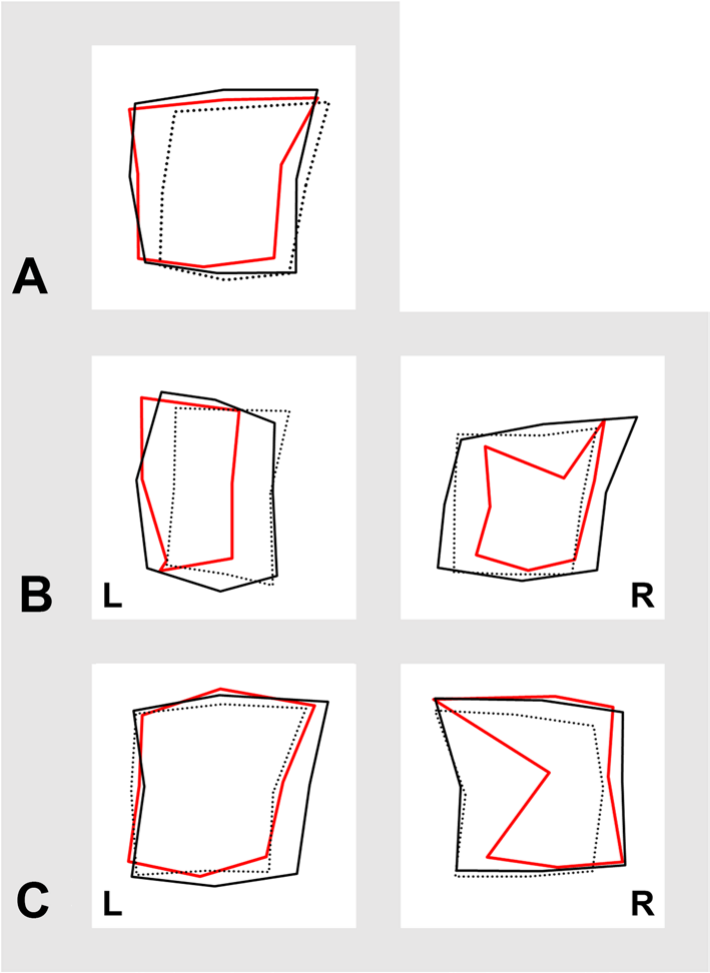
Groupwise mean *figure frames* (female participants without cognitive deficits: black polygon; male participants without cognitive deficits: black dotted polygon; patients with cognitive deficits for both sexes: red polygon). (**A**) initial reproduction with eyes closed and target matrix straight ahead. The *figure frame* overall matches the target shape. (**B**) transformation tasks: body rotation 90° in the yaw plane to the left, L, and the right, R, side. The *figure frame* shows clear deformation and decreased rectangularity in the patient cohort with cognitive impairment. (**C**) post-rotation tasks, following left-side and right-side rotation. Again, *figure frame* deformation in the cohort with cognitive impairment is visible.

No participants had to be excluded because they were unable to complete the calibration or testing paradigm. Sex differences were clearly visible with male *figure frame* typically covering a smaller area than female figures in both calibration paradigms as well as in the five pointing tasks (ANCOVA (corrected for age) mean world-based calibration area female/male F(1,73) = 5.96, p 0.02, partial η^2^ = 0.07, post-hoc difference = 19,390.66, **p**_**bonf=**_ 0.02**, Cohen’s d 0.62, t 2.44; mean retinotopic calibration area female/male F(1,73) = 15.21, p < 0.001, partial η^2^ = 0.15, post-hoc difference = 34,333.11, **p**_**bonf=**_  < 0.001***, Cohen’s d 0.99, t 3.90; mean reproduction area female/male F(1,73) = 12.10, p < 0.001, partial η^2^ = 0.13, post-hoc difference = 42,789.91, **p**_**bonf=**_  < 0.001***, Cohen’s d 0.88, t 3.48; mean transformation (non-dominant side*)* area female/male F(1,73) = 7.25, p 8.77 × 10^–3^, partial η^2^ = 0.09, post-hoc difference = 37,616.07 **p**_**bonf=**_8.77 × 10^–3^**, Cohen’s d 0.68, t 2.69; mean postrotation (non-dominant side) area female/male F(1,73) = 5.83, p 0.02, partial η^2^ = 0.07, post-hoc difference = 41,900.99, **p**_**bonf=**_0.02*, Cohen’s d 0.61, t 2.41; mean transformation (dominant side) area female/male F(1,73) = 3.05, p 0.09, partial η^2^ = 0.03, post-hoc difference = 27,395.13, **p**_**bonf=**_0.09, Cohen’s d 0.44, t 1.75; mean postrotation (dominant side) area female/male F(1,73) = 4.93, p 0.03, partial η^2^ = 0.06, post-hoc difference = 42,050.05, **p**_**bonf=**_0.03*, Cohen’s d 0.56, t 2.22))(Fig. 2). Overall, rectangularity was preserved in both male and female participants.

**Figure 3:**
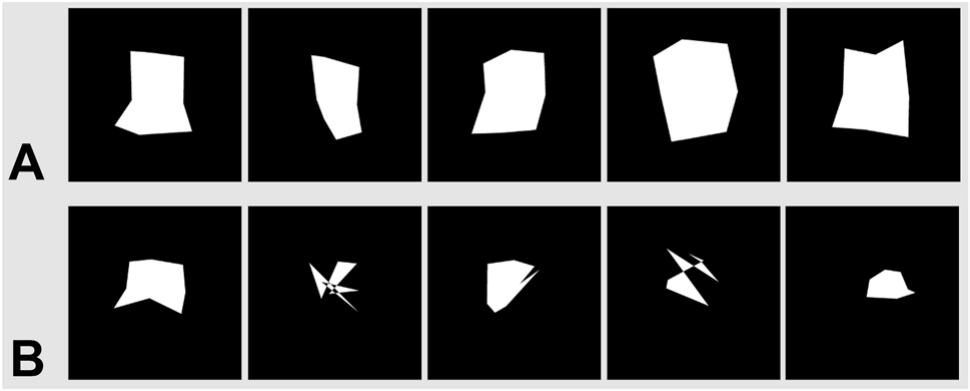
Figure frame examples of two individual patients: (**A**) An 80-year-old female with normal cognition, MoCA: 28pts; (**B**) A 76-year-old female with Alzheimer’s dementia, MoCA: 16pts. Both patients had a comparable overall azimuth angular deviation (= accuracy) in the single point analysis ((**A**) 8.87° ± 3.06°, (**B**) 9.72° ± 2.96° from their respective retinotopic calibration). From left to right: initial reproduction task, transformation to the left side, first post-rotation task back in initial position, transformation to the right side, second post-rotation task back in initial position. In patient B, the deficits became more apparent in the transformation tasks.

**Figure 4:**
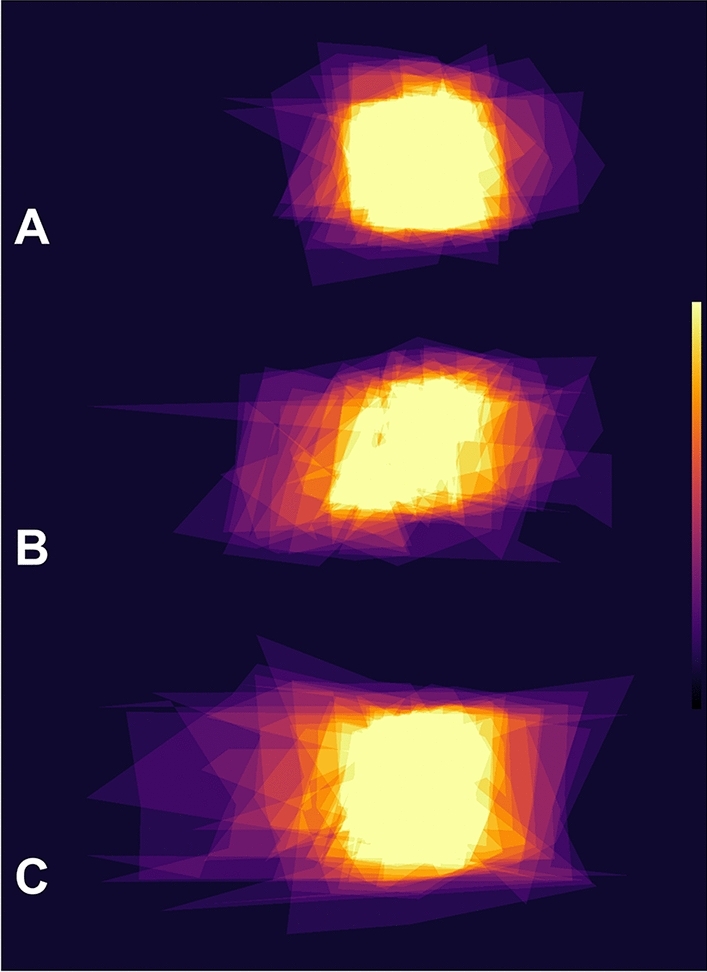
Groupwise figure frame areas of 24 individual patients, averaged over all five paradigms and intensity-coded (brighter colors = more overlap). Top row (**A**): eight patients with normal cognition, mean age 54.4 ± 21.9 years, mean MoCA: 28.9 ± 1.2pts. Middle row (**B**): eight patients with suspected mild cognitive impairment and borderline MoCA scores, mean age 70.0 ± 6.9 years, mean MoCA: 24.8 ± 0.4pts. Bottom row (**C**): eight patients with definitive cognitive impairment, mean age 76.1 ± 5.6 years, mean MoCA: 21.1 ± 1.8pts. While younger participants with normal cognition also exhibited intermittent pointing errors, patients with definite cognitive impairment made more errors (in our cohort predominantly in the azimuth (horizontal) plane), often leaving the underlying shape unrecognizable. In the patient group with borderline MoCA scores, a slight figure frame deterioration was observable. Note that age alone showed no statistically significant correlation with figure frame morphology and that the cognitive impairment was due to a variety of neurological diseases.

The data of the 22 patients with suspected cognitive impairment in the screening test were pooled for females and males because of the small total number; in the statistical analysis, we corrected for figure frame area sex differences by using the individual world-based and retinotopic calibration area as covariates. They revealed disturbed patterns of figure shapes with less resemblance to the real-world primary figure (Figs. 2B, 3, 4). This was particularly reflected by a decreased overall average diameter (i.e., the mean of all five paradigms; ANCOVA (corrected for age, world-based calibration and retinotopic calibration area) mean overall average diameter normal cognition/cognitive impairment F(1,71) = 9.30, p 3.22 × 10^–3^, partial η^2^ = 0.09, post-hoc difference = 31.22, **p**_**bonf=**_3.22 × 10–3**, Cohen’s d 0.92, t 3.05), not always reaching statistical significance in the individual paradigms themselves (ANCOVA (corrected for age, world-based calibration and retinotopic calibration area) mean reproduction average diameter difference normal cognition/cognitive impairment 28.99, **p**_**bonf=**_0.03^*^, Cohen’s d 0.68, t 2.27; mean transformation (non-dominant side) average diameter difference normal cognition/cognitive impairment 24.83, **p**_**bonf=**_0.11, Cohen’s d 0.49, t 1.63; mean postrotation (non-dominant side) average diameter difference normal cognition/cognitive impairment 28.01, **p**_**bonf=**_0.07, Cohen’s d 0.55, t 1.83; transformation (dominant side) average diameter difference normal cognition/cognitive impairment 39.48, **p**_**bonf=**_0.02*, Cohen’s d 0.74, t 2.47; mean postrotation (dominant side) average extension diameter normal cognition/cognitive impairment 34.80, **p**_**bonf=**_0.07, Cohen’s d 0.56, t 1.87). Significant area differences between normal cognition and cognitive impairment were revealed in the transformation task (body rotation) towards the dominant side (ANCOVA (corrected for age, world-based calibration and retinotopic calibration area) mean transformation (dominant side) area normal cognition/cognitive impairment F(1,71) = 4.18, p 0.04, partial η^2^ = 0.04 post-hoc difference = 36,430.37, **p**_**bonf=**_0.04*, Cohen’s d 0.62, t 2.04) but not in the other tasks. Similarly, while all subanalyses showed clear tendencies towards decreased solidity/convexity in patients with cognitive impairment and the overall average was highly statistically significant (ANCOVA (corrected for age, world-based calibration and retinotopic calibration area) overall convexity normal cognition/cognitive impairment F(1,71) = 7.79, p 6.75 × 10^–3^, partial η^2^ = 0.08, post-hoc difference = 0.07, **p**_**bonf=**_6.76 × 10^–3^**, Cohen’s d 0.84, t 2.79), only the paradigm following the rotation towards the hand-dominant side reached statistical significance by itself (ANCOVA (corrected for age, world-based calibration and retinotopic calibration area) post-rotation (dominant side) convexity normal cognition/cognitive impairment F(1,71) = 5.90, p 0.02, partial η^2^ = 0.07, post-hoc difference = 0.12, **p**_**bonf=**_0.02*, Cohen’s d 0.73, t 2.43). Figure 3 shows examples of two representative patients, one without and one with cognitive impairment.

In order to assess potential age influences, we performed a correlation analysis using Spearman’s rho in the subcohort without cognitive deficits (n = 56). Here, no statistically significant age effects were found in neither figure frame area (Spearman’s rho age/retinotopic calibration 0.16 (p 0.24), age/world-based calibration − 0.05 (p 0.72); age/reproduction 0.08 (p 0.58), age/transformation (non-hand-dominant side) 0.11 (p 0.41), age/postrotation (non-hand-dominant side) 0.16 (p 0.23), age/transformation (hand-dominant side) 0.22 (p 0.11), age/postrotation (hand-dominant side) 0.18 (p 0.19)), average diameter (Spearman’s rho age/retinotopic calibration 0.02 (p 0.87), age/world-based calibration 0.10 (p 0.47); age/reproduction 0.02 (p 0.88), age/transformation (non-hand-dominant side) 0.10 (p 0.47), age/postrotation (non-hand-dominant side) 0.16 (p 0.23), age/transformation (hand-dominant side) 0.11 (p 0.44), age/postrotation (hand-dominant side) 0.19 (p 0.15)) nor in the convexity (Spearman’s rho age/retinotopic calibration − 0.06 (p 0.68), age/world-based calibration − 0.04 (p 0.76); age/reproduction − 0.07 (p 0.61), age/transformation (non-hand-dominant side) 0.16 (p 0.25), age/postrotation (non-hand-dominant side) 0.14 (p 0.31), age/transformation (hand-dominant side) 0.07 (p 0.61), age/postrotation (hand-dominant side) 0.23 (p 0.09)).

## Discussion

In this study, we employed morphometrical analysis strategies on 2D-projections or *figure frames* of pointing vectors from a 3D-real-world-pointing test. The overall aim was centered on the feasibility of holistic analysis methods in the understanding of mental spatial representations (i.e., basic knowledge of one’s surroundings such as where an object is located in relation to an observer) and spatial conceptualizations (i.e., more complex mental representations like the relationship between an observer and multiple objects as well as their interconnections) in both healthy and diseased subjects. To our knowledge, this is the first instance in which a three-dimensional real-world pointing task has been quantified in this way. Our *figure frame* approach showed that 2D-projection and subsequent shape analysis of a 3D-pointing test can reveal additional insights into this process apart from simple angular deviation analysis (Fig. 3). Single-point analysis does not take the resulting shape into account. For example, a subject whose mental representation of the target array is enlarged (but not otherwise erroneous) compared to the real-world configuration, will systematically point with outwards directed angular deviations (e.g., 5° further left than the real-world left part of the target, 5° further right than the real-world right side). Still, since the subject’s mental shape configuration would closely resemble the target shape, the morphological properties unveiled by *figure frame* analysis would remain stable. Another subject with impaired mental shape representation might point with similar single-point angular deviations, but in their case, the 2D pointing projections and subsequent *figure frame* analyses would reveal changed morphological characteristics. On a group level, patients with definitive cognitive impairment made more large-scale errors compared to patients with normal cognition (Fig. 4A–C). One previously investigated analogy might be the misrepresentation of familiar buildings by healthy participants^21,22^; single-point analysis alone would not be able to detect overall intact, but skewed spatial representations. Based on the applied 2D-projection and subsequent shape analysis of a 3D-pointing test, there is a variety of geometrical analysis techniques available^23,24^. Our semi-direct measurement of the rectangular target already enables assessment of size, translation (in pitch-, yaw- or roll-plane) or transformation (such as scaling or shearing) as well as the aforementioned morphological aspects such as circularity, average diameter or convexity.

To account for possible anatomical confounders due to group-wise variances in shoulder-width, arm-length or joint mobility (which are irrelevant for individual analysis), we performed independent paradigm-wise analyses and corrected for calibration *figure frame* area when assessing the morphological properties of the cognitive-impairment subgroup (which included both males and females). These factors might play a role in the gender-dependent area differences already present in the calibrations with visual feedback. Both body rotation to the dominant as well as to the non-dominant side could potentially cause systematic shape alterations due to shoulder mobility: as can be seen in Fig. 2B, the *figure frame* after rotation to the hand-dominant side (left column, L) is narrower than in the null-position (Fig. 2A,C), possibly due to musculoskeletal factors when trying to point towards the far-away vertical row of target dots. This effect was more pronounced in male participants, perhaps due to commonly higher joint mobility in females^25^. However, none of the participants or patients enrolled in this study described decreased shoulder mobility or had any relevant musculoskeletal complaints.

Given the group differences in mean age, age has to be discussed as a potential confounder. Multiple studies investigated the effects of participant age on different aspects of spatial performance^26–29^. However, in the cohort of the current study no correlation between age and figure frame morphology was found in any of the subtests in participants with normal cognition. The absolute angular (azimuth) deviation, however, could be shown to increase with age in a previous study^6^. Although further research with age-matched controls is required to fully assess potential age effects, an overall intact underlying mental spatial map might persist even until high age in normal cognition, while accuracy decreases with higher age or cognitive impairment^30,31^.

Since participants were aware of the underlying square-like structure of the targets, they potentially employ some internal grid-like organization in order to point towards targets, e.g., moving straight down from “top left” to “middle left”. This assumption of a holistic mental configuration of single targets overlaps with the field of *Gestalt*-psychology^32^. Further studies could be performed with more complex shapes to differentiate between spatial localization of single targets and a simple geometrical figure. As distinct to virtual pointing tests the use of real-world pointing is a reasonable surrogate metric of measuring spatial performance because of its ubiquity in everyday life situations. Unlike other, more artificial methods of measuring participant or patient navigation abilities, this well established and lifelong optimized simple sensorimotor performance allows for more direct assessment of spatial strategies since the task itself requires no training or learning. Another advantage is revealed when testing patients with reduced cognitive function who might struggle with more complex test instructions^33^. While *Gestalt*-theories are famously hard to test in real-world applications, a simplistic, well-preserved task without prior learning might be a feasible approach when inclusion of, e.g., real-world vestibular input and actual transformation is warranted. Other more direct approaches (such as letting a participant recreate a pattern on a sheet of paper or pointing towards a computer screen) might suffice when solely investigating shape configuration^34^. Importantly, larger-scale deformations (where, e.g., middle left dot and middle right dot practically overlap) or intuitively impossible single-point orders (e.g., the left upper corner of a square can’t be closer to the right lower corner then to the left lower corner) are increasingly difficult to test in direct reproductions measurements of shape configuration.

One key difference between our testing paradigm and the large field of mental rotation research is the actual, physical whole-body rotation in the 3D-RWPT. Mental rotation has been demonstrated to involve motor circuits^35^ while vestibular stimulation itself influences mental imagery^36^. Hence, we aimed to include real-world vestibular input in our test and plan on conducting experiments in patients with bilateral vestibular loss in order to further investigate the role of the vestibular system in spatial cognition. This rotation step means that participants are required to employ spatial transformation of both their mental representations as well as their motor output^37^ to still point towards the targets. Further research might combine this real-world rotation with mental-rotation tasks to evaluate similarities and potential differences between them and help to understand the role of key neuroanatomical structures like the hippocampus^8,38,39^.

Gender-dependent differences in spatial testing paradigms have been commonly demonstrated^3,40^. Our 3D-pointing paradigm has shown similar results in a validation study: a*zimuth* (≙ horizontal) pointing performance correlated well with established 2D pen-and-paper tests and showed an age and sex dependance, while *polar* (≙ vertical) performance was more stable across sex and age^6^. It remains unclear, however, to which extent these differences are related to gender-dependent neuroanatomical patterns or sociocultural factors.

Given the heterogeneity of our patient cohort with cognitive impairment, it can only be stated that cognitive decline in general potentially not only affects the overall performance in spatial tests^41^, but also the underlying mental representation. However, further research in distinct disorders or specific atrophy patterns is needed to assess potential clinical implications. For this reason, we introduced the 3D-RWPT^6^, which, unlike other real-world navigation paradigms^42^, requires little to no patient learning and can be completed in less than 10 min, potentially paving the way for a clinical bedside test in larger patient cohorts in the future. The main purpose of the current study was to introduce and assess the feasibility of an additional holistic analysis method of the 3D-RWPT datasets.

The individual spatial skill level (as measured in tests of spatial abilities) varies widely^43^. It is therefore important to understand what factors constitute a final quantitative result in spatial performance testing. For example, wayfinding studies as one common method of spatial ability testing, might reduce a subject’s performance to parameters such as time until a destination is reached, or the number of errors made on the way. Recent work, however, showed how the employment of either allocentric or egocentric navigation strategies can change in cognitive impairment^42,44^. Similarly, pointing research thus far often focused on the end result (e.g., angular deviation between pointing vector and real-world physical directional vector), disregarding very relevant factors such as the difference between retinotopic and allocentric/world-based pointing^10,45^. Furthermore, as investigated in our study, mere angular deviation as a metric for subject pointing performance might result in similar values in both an impaired (e.g., no resemblance to the real-world target) as well as in a simply misaligned (e.g., close resemblance to the real-world target) mental representation. One recent study by He et al. could show that shortcut wayfinding (which requires an allocentric mental map) correlates with angular directional ‘pointing’ performance^46^, therefore linking these two tests of spatial performance; however, a 2D approximation was used for pointing. In our study, we utilized the raw pointing vector from a three-dimensional real world pointing task to introduce *figure frames* as a direct representation of shape configuration in a simple, well preserved sensorimotor task of spatial ability. This enables morphological qualitative analysis (e.g., “how do geometrical shape characteristics change in cognitive impairment”) and direct assessment of participants’ mental *figure frame* (e.g., “how closely does the resulting shape resemble the initial shape”), whereas future research will also focus more deeply on the quantitative end result and retinotopic vs. world-based performance in the 3D pointing test. Furthermore, distinct patient groups with clearly defined cognitive or vestibular impairment have to be included to understand the clinical relevance of this additional analysis method compared to existing tests of spatial performance.

## Data Availability

The data that support the findings of this study are not publicly available due to patient and participant privacy, but anonymized group- or paradigm-wise datasets are available on reasonable request from the corresponding author [JG].
